# Why We Eat Too Much, Have an Easier Time Gaining Than Losing Weight, and Expend Too Little Energy: Suggestions for Counteracting or Mitigating These Problems

**DOI:** 10.3390/nu13113812

**Published:** 2021-10-26

**Authors:** Katarina T. Borer

**Affiliations:** School of Kinesiology, The University of Michigan, Ann Arbor, MI 48104, USA; katarina@umich.edu

**Keywords:** overeating, inactivity, weight regain, overweight, obesity, insulin resistance, physiological barriers, psychological barriers, societal barriers

## Abstract

The intent of this review is to survey physiological, psychological, and societal obstacles to the control of eating and body weight maintenance and offer some evidence-based solutions. Physiological obstacles are genetic and therefore not amenable to direct abatement. They include an absence of feedback control against gaining weight; a non-homeostatic relationship between motivations to be physically active and weight gain; dependence of hunger and satiation on the volume of food ingested by mouth and processed by the gastrointestinal tract and not on circulating metabolites and putative hunger or satiation hormones. Further, stomach size increases from overeating and binging, and there is difficulty in maintaining weight reductions due to a decline in resting metabolism, increased hunger, and enhanced efficiency of energy storage. Finally, we bear the evolutionary burden of extraordinary human capacity to store body fat. Of the psychological barriers, human craving for palatable food, tendency to overeat in company of others, and gullibility to overeat when offered large portions, can be overcome consciously. The tendency to eat an unnecessary number of meals during the wakeful period can be mitigated by time-restricted feeding to a 6–10 h period. Social barriers of replacing individual physical work by labor-saving appliances, designing built environments more suitable for car than active transportation; government food macronutrient advice that increases insulin resistance; overabundance of inexpensive food; and profit-driven efforts by the food industry to market energy-dense and nutritionally compromised food are best overcome by informed individual macronutrient choices and appropriate timing of exercise with respect to meals, both of which can decrease insulin resistance. The best defense against overeating, weight gain, and inactivity is the understanding of factors eliciting them and of strategies that can avoid and mitigate them.

## 1. Introduction

Since 1975, the worldwide rate of obesity has tripled [1], and in 2018, 42.4% and 31.1% of adult Americans were obese and overweight, respectively [2]. The unremitting increase over the past half century in the rate of overweight and obesity and their associated disabilities in U.S.A and other developed countries suggests that growth of obesity has epidemic features that require urgent mitigation. Without an attenuation of current weight gain trends, 1.35 billion people worldwide will be overweight and 573 million obese by 2030 [3]. Among the health problems usually listed as being associated with overweight and obesity are cardiovascular disease (CVD), hypertension, type 2 diabetes (T2D), hyperlipidemia, stroke, certain cancers, sleep apnea, liver and gall bladder disease, osteoarthritis, and gynecological problems [4,5]. There are also psychosocial consequences of obesity such as experience of weight stigma or perceived weight discrimination. These are associated with depression, anxiety, bulimia, body dissatisfaction, and low body and self-esteem [6,7,8]. Medical and surgical interventions against obesity have predominantly been applied to the most obese but have not been uniformly successful or without side effects. Behavioral interventions are hindered by psychosocial factors such as ingrained personal and family habits and societal customs.

The data presented in this review are based in part on PubMed and Google Scholar search for relevant supporting articles as well as the author’s research findings and views on the regulation of energy balance in humans [9,10,11,12,13,14,15,16,17,18,19]. There is insufficient understanding in four areas connected to obesity and its causes. The first one is inadequate understanding of the details of how obesity generates serious health problems. The second one is a general unawareness of the limitations of human physiology in control of weight gain and loss. The third one is how our misguided psychology toward eating contributes to overeating. Additionally, the fourth one is insufficient recognition of the features of developed societies that hinder efforts to control our weight.

An explanation of how obesity causes hormonal dysregulation of energy balance that results in insulin resistance, the key cause of obesity-linked pathologies, will be described first. In addressing the limitations of human physiology in control of weight gain and loss, evidence will be provided for the genetic basis of human appetite, predisposition for accumulation of fat, and absence of a negative-feedback mechanism of energy regulation. Regarding human psychological attitudes toward food, human seeking of palatable food, undisciplined eating, social facilitation of overeating, and opportunistic eating when overabundance of food is available at low cost, is examined. In addressing the societal factors that hinder human efforts to control body weight, data will be presented on the role of technological labor-saving developments, policies on dietary intake and housing patterns, and the efforts of profit motives of the food industry in promoting high-density palatable foods.

## 2. How Obesity Generates Serious Health Problems

The principal health problem associated with obesity is induction by obesity of insulin resistance. Insulin resistance is a consequence of disturbed endocrine regulation of insulin, the key controller of body energy storage and mobilization. Insulin is secreted in response to circulating glucose and amino acids to produce four actions, facilitate uptake of these nutrients by muscle and some other tissues, increase the metabolic utilization of blood glucose thus producing a hypoglycemic effect through these two actions, promote energy storage primarily through stimulating synthesis of glycogen in the liver and triglyceride in the adipose tissue, and block the breakdown, release, and metabolism of metabolic fuels from their inert storage forms. These four actions characterize insulin as a key mediator of energy storage. It is less well recognized that the hormone leptin significantly contributes to meal-associated regulation of energy balance as well. While its release from the subcutaneous white adipose tissue (WAT) is universally recognized, it is less well known that leptin is also released from the stomach during meal eating [20]. Its meal-associated secretion and actions counter-regulate insulin secretion and actions. Insulin stimulates leptin release during food intake, and leptin then restrains all four of insulin’s actions as well as insulin release [12]. Leptin indirectly restrains the energy storage actions of insulin by promoting lipolysis and stimulating lipid metabolism. This short-term meal-associated endocrine regulation of energy balance is disrupted in obesity by development of resistance of peripheral tissues to both insulin and leptin.

**Figure 1:**
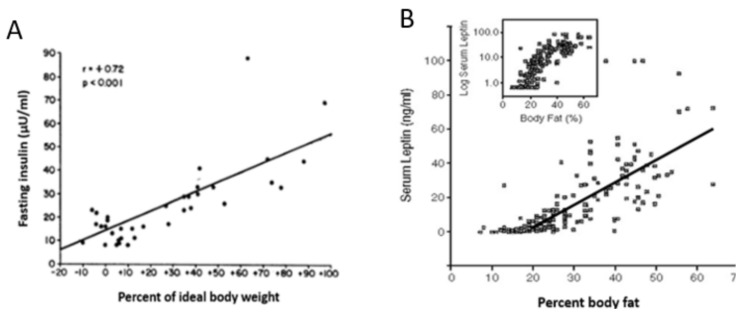
The positive correlation between fasting serum insulin (**A**) and fasting serum leptin (**B**) as a function of percentage of body weight or body fat. Adapted from Bagdade 1968 for insulin and from Considine et al., 1996 for leptin.

Increased insulin and leptin resistance in obesity [21,22] is manifested as a proportional rise in fasted and stimulated blood concentrations of both hormones [23,24] as a function of increased body fat (Figure 1). The level of insulin resistance in obese humans is measured by the disposition index (DI) [25]. The DI is a product of insulin sensitivity and the amount of insulin secreted to a given blood glucose load. The relationship is parabolic and shows that little insulin secretion is needed to exert its four actions when insulin sensitivity is high, but that a high insulin concentration is required when insulin sensitivity is low.

Therefore, in obese persons, insulin-resistant peripheral tissues require high basal and stimulated insulin concentrations.

How insulin resistance develops at a cellular level can be explained by the fundamental relationship between the number of insulin receptors on the hormone’s target cells such as adipocytes, and the concentration of insulin needed to produce a biological response. A given biological response requires stimulation of a minimum density of receptors. The greater the density of receptors, the less hormone is needed. With lower body weight and reduction in the volume of each adipocyte, the density of insulin receptors on its membrane significantly increases, producing “spare receptors” in excess of what is needed to produce the biological response. That is why weight loss that reduces the size of adipocytes produces enhanced insulin sensitivity [12,26]. With obesity, adipocyte size hypertrophies and the density of insulin receptors on adipocyte membranes decreases [27]. This then necessitates higher concentrations of insulin to elicit the needed biological response. This fundamental role of hormone–receptor interactions in the context of body energy repletion and depletion explains why concentrations of insulin and leptin rise in obese persons (Figure 1) and animals and why insulin becomes less effective in suppressing hyperglycemia and hyperlipidemia.

The key health damages from insulin resistance include hyperglycemia and compensatory hyperinsulinemia that result from insulin resistance [28]. Obesity thus drives the progression from insulin resistance, glucose intolerance to pre-diabetes and T2D. The incidence of T2D has increased in parallel with the rise in obesity, from 4.4 million or 2.4% of US population in 1970s [29] to 34.2 million or 10.5% of the population in 2021 [30]. The global prevalence of diabetes in 2010 was 284 million people worldwide, constituting approximately 6.4% of the world population; and in 2018, it was estimated that there were 500 million diabetics [31]. The projections for 2030 show the T2D prevalence to comprise ~7.7% of the world population [32]. Hyperglycemia and hyperinsulinemia also lead to glycation of circulating proteins and the formation of advanced glycation end products associated with pathological oxidative stress [33]. Additional detrimental health consequences of insulin resistance include ectopic fat deposition in tissues other than WAT. When adipocyte capacity for hypertrophy is exceeded, high insulin concentrations drive fat deposition in the liver [34,35], the pancreas [35], the muscle [35], and the kidney [36]. Ectopic fat deposition causes lipotoxicity as a byproduct of insulin resistance [37]. Insulin resistance is sustained and increased through a vicious cycle. High free fatty acid (FFA) concentrations result from diminished suppression by insulin of their mobilization from WAT and directly interfere with insulin signaling in the muscle [38].

## 3. Limitations of Human Physiology in Controlling Weight Gain and Loss

*Point 1: There is no negative-feedback regulation of body weight*. An important fact that is not universally accepted is that the apparent stability of adult body weight in approximately half of non-obese American population is not a consequence of a regulated process based on negative-feedback compensations. This concept is unsettling as it alerts us that there is no automatic inborn mechanism for maintenance of healthy body weight. A number of hypotheses have unsuccessfully attempted to explain this apparent weight stability as representing a weight setpoint based on feedback adjustments in spontaneous food intake and physical activity [39,40]. As we are witnessing in the USA and other developed countries, there is no apparent negative feedback to prevent weight gain in overweight and obese individuals, although there is a robust increase in hunger and even energy-saving reduction in metabolic rate [41] with any significant weight gain. The popular formulation of the setpoint hypothesis is based on the expectation that the negative-feedback signal that encodes decreases in adipose tissue mass is a reduction in the circulating concentration of the hormone leptin released from the subcutaneous WAT [42]. This hypothesis was triggered in part by the observation that leptin injections reduce hunger and produce weight loss in obese humans genetically unable to produce leptin [43]. The expectation that the same relationship operates in neurologically normal humans did not materialize. In a large trial where obese individuals were injected with several doses of leptin ranging from sub-threshold to supra-physiological levels, there was no effect on appetite or weight loss [44]. In addition, leptin concentrations rise in both humans and animals parallel with the rise in obesity (Figure 1), a clear demonstration that this hormone does not operate as a weight-normalizing negative-feedback signal. It is more likely that injected leptin’s effectiveness in causing weight loss in congenital leptin insufficiency is due to its lipolytic properties and its role in counter-regulating the obesifying insulin action [12].

**Figure 2:**
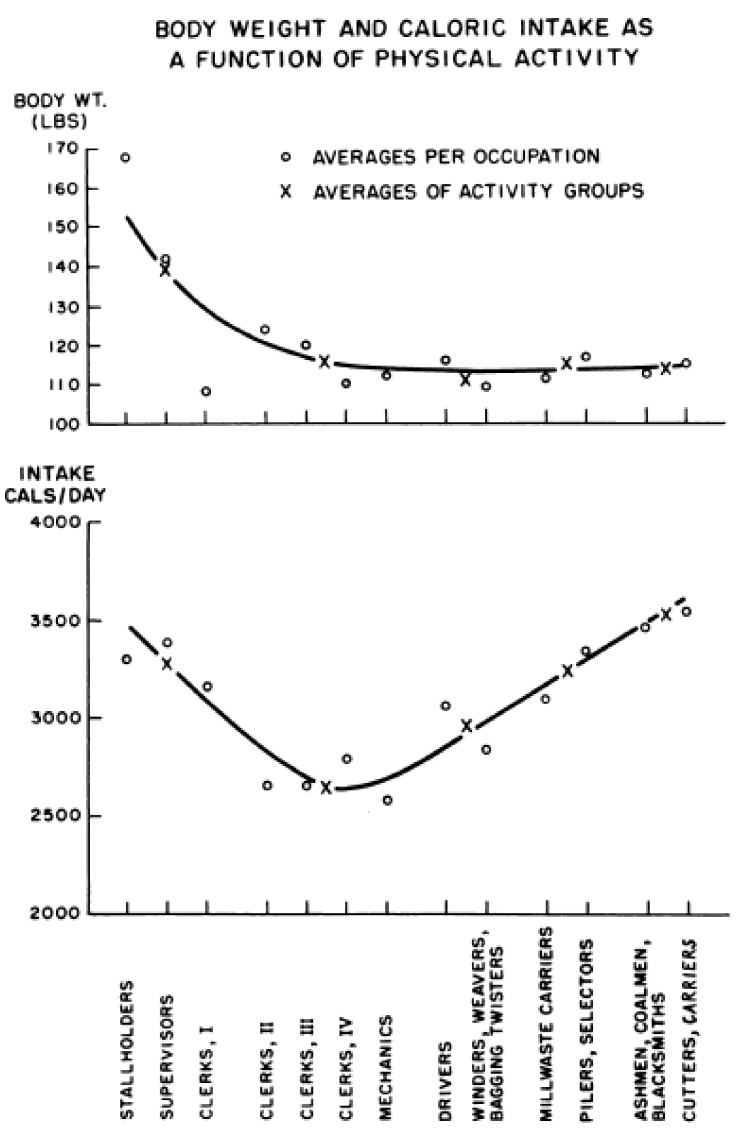
The relationship between body weight and food intake as a function of increasing physical work. From Mayer et al., 1956. Reproduced with permission.

*Point 2: the relationship between body fatness and the motivation to expend energy is non-homeostatic in humans as well as in animals [11]*. This concept can be best visualized in Figure 2 showing the results of a 1956 study by Jean Mayer [45].

**Figure 3:**
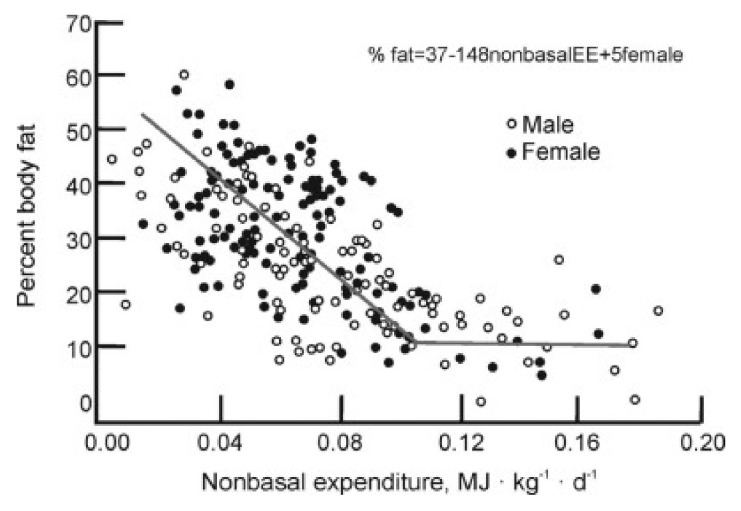
Inverse relationship between voluntary daily energy expenditure in healthy humans as a percent of body weight. Modified from Rising et al., 1994 and Schultz and Schoeller, 1994.

In this observational study, adult West Bengal Indians who engaged in occupations that required between light and medium through very heavy physical work maintained their weight by upward adjustments in their food intake in parallel with their energy expenditure. It is not difficult to understand that this relationship reflected increased hunger as the energy expenditure exceeded the usual meal sizes. Where the clue for dietary obesity lies is in the non-homeostatic increase in food consumption as the level of physical activity declines between light work and sedentary condition. Within this inadequate physical activity range, characteristic of at least one half of American population, there is a counter-intuitive reciprocal relationship between weight gain and decreased motivation to be physically active [11]. This relationship is apparent in a survey of voluntary energy expenditure in adult Americans as a function of body fat [46,47] (Figure 3).

**Figure 4:**
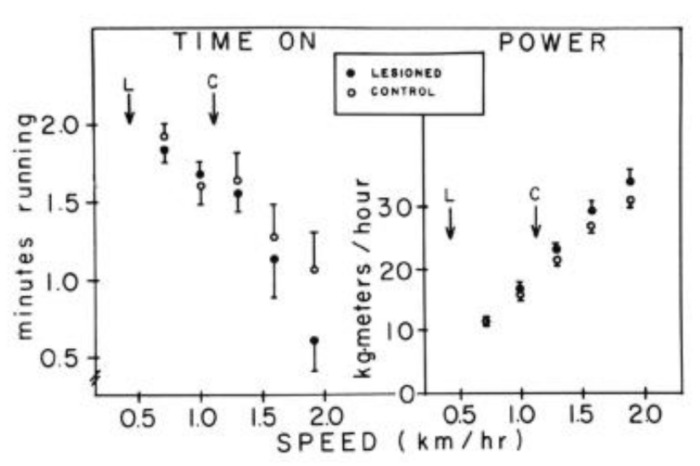
Obesifying septal lesions in golden hamsters (Mesocricetus auratus) reduced spontaneous disc running by 82%. However, when forced to run on a motorized treadmill with electrified off-ramp grid as a negative reinforcement, obese hamsters ran as long (left panel) and as rapidly (right panel) as neurologically intact hamsters. From Borer et al., 1983. Reproduced with permission.

Highest non-basal or voluntary energy expenditure was recorded in individuals containing the least amount of body fat (8 to 20%, right end of Figure 3). As the body fat increased between 10 and 60% of body weight (left end of Figure 3), voluntary energy expenditure proportionally decreased. The greater the weight gain, the lower the motivation to move. This is an involuntary innate relationship, and not based on simple Newtonian relationship requiring greater effort to move a heavier body as shown by a simple study comparing the speed and duration of forced physical activity in normal-weight hamsters and experimentally-induced obese hamsters [16]. By having both groups run on a motorized treadmill negatively reinforced for failure to run, the two groups displayed identical capacity to run at increased speeds and comparable endurance (Figure 4).

It is reasonable to conclude from the presented data that humans and animals eat non-homeostatically when insufficiently active.

**Figure 5:**
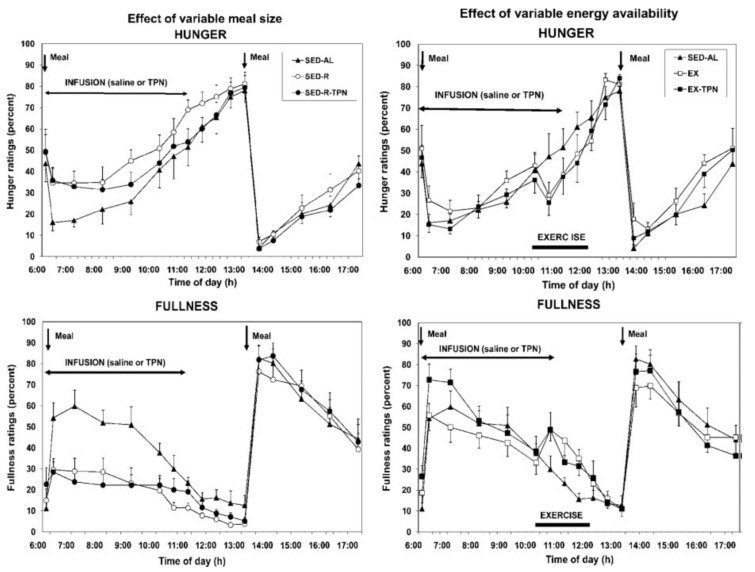
The effects of variable meal size (left) and energy availability after exercise (right) on the psychophysical ratings of hunger (top) and fullness (bottom) in 10 postmenopausal women subjected to a sedentary trial with a large 500-kcal morning meal (SED-AL), or a small 100 kcall morning meal (SED-R, left panel), or to 2 h of moderate-intensity exercise after a large morning meal (EX), and iv nutrient infusion (TPN) as a replacement of energy withheld from a morning meal (SED-R-TPN) or expended through exercise (EX-TPN, right panel). From Borer et al., 2009. Reproduced with permission.

*Point 3. Hunger and satiation are mediated by oral and gastrointestinal signals and not by circulating metabolites or hormones*. There have been repeated attempts to link hunger either to changes in circulating metabolites such as glucose, ketones, or FFAs, or to hormones such as leptin and insulin (the putative satiety hormones [42,43]) or to ghrelin as the putative hunger hormone [48]. Neither hypothesis has withstood testing. The assumption that circulating metabolites or nutrients influence hunger was contested in two studies [18,49]. In the first one [18], hunger was measured in response to different size meals (100 vs. 500 kcall) taken by mouth but with intravenous supplementation of small meals with parenteral nutrients. Hunger was also tested when the large meal was combined with exercise which depleted close to 90 percent of ingested calories. Again, the energy shortfall was compensated by intravenous infusion of nutrients. As Figure 5 clearly shows, only the size of meals ingested by mouth and processed by the gastrointestinal tract influenced hunger and satiation. With the 100 kcal meal, the fullness rating was lower (Figure 5, bottom left panel), and hunger much greater (top left panel), than with the 500 kcal meal. Intravenous infusion of nutrients of similar composition to that of orally eaten meals, did not influence either the hunger or satiation. In a similar vein, calories expended by exercise were not detected and reflected in increased hunger (top right panel) or in reduced satiation (bottom right panel). Only a variation in the size of the meal eaten by mouth and processed through the gastrointestinal tract affected the magnitude of hunger and satiation and remained unaffected by supplementation of intravenous calories or by exercise energy expenditure.

**Figure 6:**
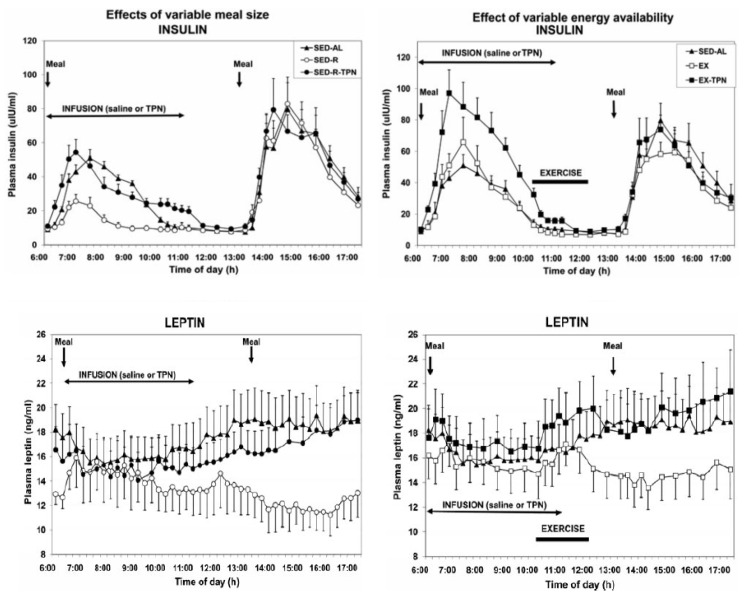
Insulin concentration (top panels) and leptin concentration (bottom panels) were proportional to the concentration of circulating nutrients derived from ingested meal and infused parenteral nutrition in sedentary condition (left panels) or in response to exercise (right panels). From Borer et al., 2009. Reproduced with permission.

The second study [49] confirmed that increases in circulating metabolic fuels affected by exercise in fasted state, or by calories absorbed from meals during post-meal exercise, had no impact on hunger and satiation ratings. Finally, the confirmation that satiation reflects the volume of ingested food and not their caloric content was demonstrated in a study where healthy volunteers were provided for 11 weeks with identical diets that differed only in fat content, a higher-fat diet containing 30 to 35% fat, or lower-fat diet with 20 to 25% fat [50]. Both groups consumed approximately the same daily volume of food (between 1400 and 1450 g) but did not adjust the quantity eaten to account for the difference in dietary fat content. As a result, the body weights of the two groups diverged. Finally, while hunger and satiation responded only to quantity of orally ingested food, the putative satiety hormones insulin and leptin responded to the state of all circulating fuels, the absorbed food that was eaten, infused parenteral nutrition, and calories lost through exercise [18] (Figure 6). However, the concentrations of these hormones, which tracked fuel availability in circulation, did not affect hunger or satiation.

**Figure 7:**
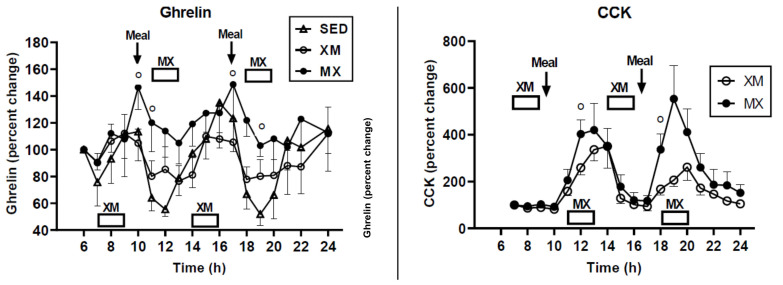
Concentrations of putative hunger hormone ghrelin (left) and validated satiety hormone cholecystokinin (CCK, right) increase in response to meals that preceded exercise (MX) but not to exercise (XM) when it preceded eating. From Borer et al., 2021.

Similarly [49], there was no effect on hunger or fullness ratings during postprandial periods when both the putative hunger hormone ghrelin [48] (Figure 7, left panel) and the validated satiety hormone cholecystokinin [51] (Figure 7, right panel) showed equivalent and parallel postprandial rises and declines without affecting the pattern of hunger and satiation.

Given that the above data indicate that hunger is not caused by ghrelin, and that fullness is not a consequence of fluctuations in plasma insulin, leptin, and CCK, the probable source of hunger and satiation resides within the gastrointestinal tract. More than a hundred years ago, gastric contractions were singled out as the most plausible stimulus for hunger in classical experiments of Cannon and Washburn [52]. In these impressive studies, now obliterated by an emphasis on the involvement of hypothalamic feeding circuitry, a strong correlation was found between reports of hunger pangs and stomach contractions measured in volunteers supplied with instrumented intragastric water balloons. Satiation and fullness have been attributed both to the stomach distension as well as to hormonal consequences of intestinal nutrient digestion and absorption [53,54]. Thus, the basic blueprint of human hunger and fullness mechanism does not fundamentally differ from that uncovered in a blowfly [55]. The insect seeks food when its crop is empty, and stops eating when the crop is full, just as humans become more spontaneously active when their stomachs are empty, and quiescent when they are full [56,57].

*Point 4. Stomach size adapts to habitual volumes of consumed meals.* If gastrointestinal signals are likely to guide our nutrient consumption by alerting us to hunger and satiation, the volume of food eaten relative to stomach size should play an important role. While the size of an average stomach is thought to match that of a grapefruit, there is substantial evidence that it will increase if it is habitually filled to overcapacity. Therefore, fullness signals may be attenuated if the stomach increases beyond the normal size. Individuals who engage in excess food binging have a larger stomach volume than individuals who do not, and the effect is related to binge-eating and not to their body weight [58]. This effect is amplified in people engaging in hot-dog overeating competitions. They develop up to 700% greater capacity to eat rapidly and store greater quantities of food in their stomachs which require extended periods of time to get digested [59]. The anatomical changes associated with binging, gorging and purging, have recently been found to affect brain circuits mediating hunger and satiation [60]. Conversely, a year-long total fast that resulted in massive weight loss was reported to produce no substantial weight regain rebound 5 years later [61], suggesting operation of appetite control through possible stomach atrophy due to disuse. Surgical approaches toward solving the obesity problem to a large extent include reductions in stomach size. Various forms of gastrectomy, ranging from banding, removing portion of greater stomach curvature (sleeve gastrectomy), to Roux-en-Y procedure, all share the features of reduced hunger associated with smaller stomach size [62,63]. Along with the already presented data, reductions in hunger in response to surgical reduction in stomach size provide additional support for the role of gastrointestinal signals in the control of hunger and satiation.

*Point 5. Adaptive thermogenesis and increases in insulin sensitivity and hunger hamper loss of body fat*. An inconvenient finding for most individuals trying to lose weight is that deliberate weight loss through food restriction, with or without added exercise, is regained in the matter of months or years [64]. Three powerful innate physiological defenses interfere with the maintenance of weight loss, whether it is achieved from the obese or healthy weight level. The first one, called adaptive thermogenesis, consists of persistent reduction in resting metabolic rate (RMR) [65], the second one is increased hunger, and the third one is enhanced efficiency of energy storage. Adaptive thermogenesis was studied in individuals who lost substantial amounts of body weight and body fat in attempts to win “The biggest loser” televised competition. In comparison with the weight loss produced by Roux-en-Y gastrectomy, with both producing a loss of between 40 and 49 kg, respectively, and with a smaller 16% loss of lean body mass in the televised competition [66], RMR decreased more in both groups than expected based on measured body composition changes. The magnitude of this metabolic adaptation was correlated with the magnitude of energy imbalance and the decrease in circulating leptin. The persistence of this adaptive thermogenesis response was revealed after 6 years when 16 of the “Biggest Looser” competitors were re-tested [41]. They regained 41 kg of 58 kg lost, but their RMR remained 500 kcal/day below the expected level, representing an innate physiological defense against weight loss. This response is currently interpreted as an evolutionary defense against a reduction in total daily energy expenditure [66]. In this view, higher energy expenditure due to increased body mass is sustained with higher energy intake, but any reduction in intake, increase in exercise energy expenditure, and reduction in body mass is compensated by a reduction in RMR. The other two innate processes that interfere with weight-loss maintenance and promote weight regain are a large increase in the efficiency of energy utilization and increased hunger. Through reduction in the size of adipocytes and increase in the density of insulin receptors on their cell membranes, weight loss significantly increases insulin sensitivity and thus the effectiveness of its four actions. Insulin now more powerfully promotes nutrient uptake, glycogen and fat synthesis, and blocks fuel store degradation. Fasting plasma leptin is now reduced to very low levels (Figure 1) which reduce satiation and promote hunger. This role of leptin as a “starvation” hormone has been demonstrated by administering the hormone to subjects who have experimentally undergone a 10% weight loss. Leptin administration suppressed their hunger and helped maintain their weight loss [67].

**Figure 8:**
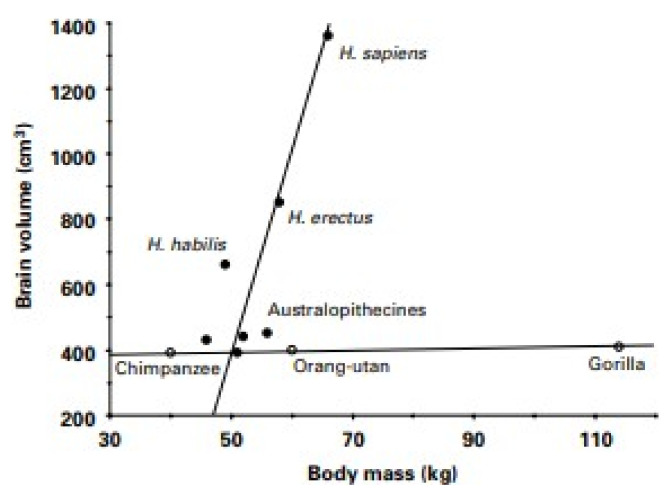
Increases in hominid encephalization. From Wells, 2006. Reproduced with permission.

*Point 6. Evolutionary burden of human large capacity and predisposition for body fat gain and storage.* Among primates, some of whom average between 5 and 10% body fat, humans have exceptionally large fat depots, between 12 and 23% in normal-weight men, and between 24 and 34% in women [68]. Increased capacity to store fat is hypothesized to have co-evolved with the high degree of encephalization in the genus Homo [69], which became exponential over the past two million years to triple human brain size relative to other primates (Figure 8).

It is speculated that reduced energy cost of bipedal locomotion and seasonal variability in energy resources in the terrestrial savannah environment drove the coevolution of physiological buffering against energy deficit in the form of fat storage and of cognitive buffering in the development of capacities to find new food sources. Increased fat storage also was necessary to support the evolution of larger brain size as human brain consumes between 20 and 25% of RMR. Natural selection for increased fat storage has also increased the capacity of ancestral women to bear multiple children in rapid succession compared to several-year-long intervals between infant births in other primates. Increased adiposity of females has provided support for the energy cost of pregnancy, lactation, and the feeding of multiple children.

### Suggestions for How to Deal with Limitations of Human Physiology in Controlling Weight Gain and Loss

The six mentioned limitations of human physiology cannot be directly counteracted because they are genetically programmed and beyond direct voluntary control. However, awareness of their operation can guide human behavior. All six provide a cautionary message that the choice of how much we eat begins by understanding that any automatic feedback will only guard against body weight and fat loss, but not against body weight and fat gain. Therefore, we must be sensitive to gastrointestinal signals of hunger and fullness. Overeating beyond signals of fullness can produce adaptive enlargement of stomach size attenuating the gastric signals to stop eating. Once we gain excessive weight we have to contend with reduced metabolic rate and other automatic defenses against weight loss.

## 4. Limitations of Human Psychology in Controlling Weight Gain and Loss

We have a better control over our behavior when it comes to consciously selecting what and how much we eat than was the case with physiological limitations to gaining and losing weight.

*Point 1: We can consciously control our seeking of palatable odors and tastes*. Although we have an inborn preference for the sweet taste, and dislike for bitter and sour taste manifested at birth [70], we discriminate between seeking food when we are hungry and desiring food that smells and tastes good. Hunger produces a deliberate “wanting” of a food reward, while our motivation for palatable odors and tastes represents our “liking” or desiring food reward [71]. Substantial orbitofrontal and insular cortical and limbic circuits have been identified as a substrate of the hunger motivation in response to negative energy balance and inadequate consumption of food. Interspersed within this circuitry, and centered in the mesolimbic nucleus accumbens, are neural substrates of hedonic motivation that can amplify human seeking of palatable tastes and odors which operate with dopamine and opioids as neurotransmitters. Beyond the motivation for palatability, the appeal of food is enhanced also by its variety, which can increase intake by as much as 29% [72]. We acknowledge our liking of palatable food and of its variety through the practice of gastronomy. Humans practice gastronomy and accommodate the desire for palatability daily in the way they sequence palatable foods in their meals to progress from salty and savory to maximally palatable sweets at the end.

**Figure 9:**
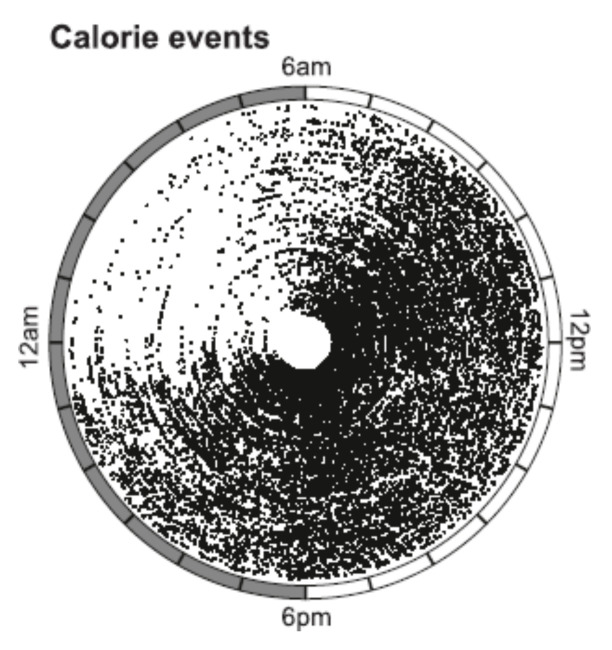
Calorie-containing (R5 kcal) (D) ingestion events of each individual plotted against the time of day (radial axis) in each concentric circle. From Gill and Panda, 2015. Reproduced with permission.

*Point 2. Humans consume a number of unnecessary meals and snacks in a day.* Using a smart-phone application that allowed recording of feeding episodes greater than 5 kcal, a recent study revealed a chaotic human feeding pattern that does not universally conform to the assumed three-meals-a-day pattern [73]. One hundred and fifty six healthy men and women were monitored for 3 weeks, and 20,800 food-intake events were recorded (Figure 9). Average estimated caloric intake was 1950 kcal, in excess of the estimated 1230 kcal maintenance energy level.

The remarkable feature of the study was showing that the number of individual food-intake events ranged between 3 and 11 per day (Figure 9) and extended over a 19 h wakeful period with no feeding occurring only during the 5 h sleep period between 1:00 and 6:00 h. Only 25% of caloric intake occurred before noon. The percentages of total calories consumed after 6 pm, 9 pm, and 11 pm (and before 4 am of the next day) were 37.5%, 12.2%, and 3.9%, respectively. A subset of 8 subjects, who exhibited greater than 14 h spontaneous eating durations, were recruited for a 16-week intervention of restricting their eating within a self-selected 10–12 h time period. The result was a 3.25 kg weight loss, 1.15 kg/m^2^ reduction in BMI, and improved assessment of sleep satisfaction, hunger at bedtime, and energy level.

*Point 3. Humans eat more in company of others*. Social facilitation of food intake, a term coined by John De Castro [74], is a phenomenon humans share with a number of other animals such as dogs [75] and chickens [76]. Social facilitation implies that the amount eaten by humans in spontaneously ingested meals is positively correlated with the number of other people present. Socially facilitated food intake can increase by 44% and is related to the duration of meals and not to an increase in hunger. Others in the group can be strangers such as when eating in a restaurant [77], or they may represent virtual company as in food commercials seen on television [78]. Another variable in social settings that increases the amount eaten is matching the speed of eating seen in other people [79].

*Point 4. Opportunistic eating as a function of food quantity*. Humans eat more food when it is provided in larger quantities. This takes several forms: (1) more is eaten with larger food portions whether presented during meals [80] or available in packages [81]; (2) more is eaten from larger containers [82]; and more is eaten in response to advantageous price incentives offered by fast-food companies. Higher-calorie combination meals in fast-food restaurants offer significantly more calories per dollar compared to regular meals, suggesting there is a strong financial incentive for consumers to ‘upsize’ their orders [83].

### Using Psychology for Controlling Overeating

Psychological factors contributing to overeating can be managed by first understanding their causes. Predilection for palatable food can be rationally controlled by enjoying good tasting food without allowing its available quantity to dictate how much we eat. We need to expect and prepare for oversized servings in restaurants, food packaging that misrepresents the contained calories, and social situations or celebrations that promote food overconsumption. Simply understanding that we tend to eat unnecessary meals and snacks over an extended wakeful period has another clear solution by establishing a time-restricted feeding pattern [84]. Limiting food intake to a 6 to 10 h daily time period reduced food intake, weight gain, and caused fat loss without eliciting excessive hunger both in obese and diabetic mice [85] ovariectomized mice [86], in obese [87] and pre-diabetic humans [88], and in obese postmenopausal women [89], and therefore can be implemented to prevent overeating and excessive weight gain.

## 5. Features of Developed Societies That Hinder Efforts to Control Our Weight

Large human brains have contributed to development of technologies, structured the built environments, instituted societal policies, and fostered economic growth, the first two of which have reduced the need for physical work and the other three have facilitated food overconsumption. Labor-saving devices have brought humanity, especially in developed countries, to the non-homeostatic range of interactions between weight gain and physical activity (left part of Figure 2).

*Point 1. Development and introduction of electricity and gas-powered appliances and modes of transportation have had a major labor-saving effect*. Discussion of the likely magnitude of physical effort required of our Pleistocene ancestors to run down wild ungulates for meat would be futile from the perspective of our current lifestyle. However, a comparison with a contemporary population that rejects the use of electricity in their lifestyle can better remind us of the ways we have replaced manual work with mechanized devices. The population in question is an Old Order Amish community in Canada studied by David Basset in 2004 during their planting season [90]. Table 1 summarizes current mechanized substitutions for the manual work done by Amish.

While caloric values can be quoted for some manual work that is in contemporary lifestyle replaced by appliances (e.g., moderate effort for a 74 kg person requires approximately 3 to 6 kcal/min and heavy effort approximately 10 to 20 kcal/min), the scale and duration of work possible with contemporary mechanized devices vastly exceeds what is possible by physical work done by an individual. Old Order Amish men expended on the average 10 h/week of vigorous physical activity (PA), 42.8 h/week of moderate PA, and 12 h/week of walking which entailed an average of 18,425 steps per day. Average daily energy expenditure was estimated at 3100 kcal/day in men and 1850 kcal/day in women. Amish women who cared for their large families, did the domestic work, and helped with farming, reported engaging in 3.4 h/week of vigorous activity and 39.2 h/week of moderate activity. Their daily step count was 14,196. As can be expected from these levels of energy expenditure, Amish lifestyle placed them in the homeostatic range of body-weight relationship presented on the right side of Figure 2. The incidence of obesity in their community was only 4% and of overweight 26%. 

Of contemporary labor-saving devices, the effect on body fat of having a car and driving as a mode of transportation has attracted the most attention. A number of cross-sectional studies have documented that car use for transportation was associated with increases in body weight and waist circumference. A dose-dependent relationship was found between the length of time spent driving and odds of being overweight or obese [91]. Driving 840 to 1680 min/week doubled these odds compared to driving less than 210 min/week. A 3-fold increase in the likelihood of overweight was recorded among insufficiently active individuals who drove 210 to 420 min/week. In contrast to the cross-sectional studies which could only uncover an association between the quantity of driving a car and weight gain, one of the more convincing studies took advantage of the low 16% level of car ownership in China at the turn of the 21st century to examine changes in body weight in parallel with increases in. car ownership [92]. Over the 8 year observation period, during which 14% of households acquired a motorized vehicle, the odds of being obese were 80% higher for men and women in households who owned a car compared to those who did not. Men who acquired a vehicle experienced a 1.8 kg greater weight gain and had two times the odds of becoming obese compared to those who did not. Conversely, engaging in active transportation, walking or riding a bicycle to work, reduced the odds of becoming obese [93]. Among the 9856 individuals in Sweden, those who rode a bicycle or walked to work had a 38% lower odds of being overweight and obese compared to the car-driving subjects.

*Point 2. The built environment in economically developed countries does not usually facilitate active modes of transportation.* Studies of the relationship between the types of built environment and weight gain indicate that multiple-use environments (a mixture of residential, commercial, office, and institutional) promote active transportation of walking and bicycling more than single-use environments which necessitate the use of cars [94]. Each quartile of increase in the multiple-use built environment decreased the likelihood of being obese by 12.2%. Each additional kilometer walked per day was associated with a 4.8% reduction in the likelihood of obesity.

*Point 3. Government policies encouraging specific patterns of macronutrient intake in some cases favor overeating and weight gain*. As was outlined earlier, postprandial hyperglycemia (>7.8 mmol/L) is detrimental to health and is a diagnostic symptom of T2D where it is prevalent throughout the day [95]. Both persistent hyperglycemia as reflected in elevated hemoglobin A1c and reactive hyperinsulinemia [96,97] reflect insulin resistance and have been associated with adverse coronary heart disease (CHD) outcome [98,99,100] and with increased mortality risk [96,97]. The association of hyperglycemia and hyperinsulinemia in T2D with obesity is firmly established in the term diabesity [32] and the fact that both can be reduced with weight loss [28,101]. However, it remains controversial how much the rise in T2D incidence from 4.4 million or 2.4% of the US population in 1970s [29] to 34.2 million or 10.5% of the population in 2021 [30], was facilitated by a 30.5% increase in daily carbohydrate consumption from 213 g per day of daily calories in 1965 to 278 g per day or 51% in 2011 [102]. The current carbohydrate consumption falls within the 45 to 65% of daily calorie range recommended in 2010 by Departments of Agriculture and Health and Human Services (DAHHS) [103]. This outdated policy was influenced by the hypothesis posited in 1986 that high dietary cholesterol was the principal cause of CHD [104]. This prompted DAHHS in 2010 to recommend a shift in macronutrient selection away from lipids in favor of high carbohydrate intakes [103]. A study comparing the effects of eating a 60% carbohydrate diet to a 30% carbohydrate diet on glycemia, insulin responses, and HOMA-IR assessment of insulin resistance demonstrated that lowering the carbohydrate component of the meals improved insulin sensitivity by approximately 30% within a 3-meal exposure to the changed diet [19].

**Figure 10:**
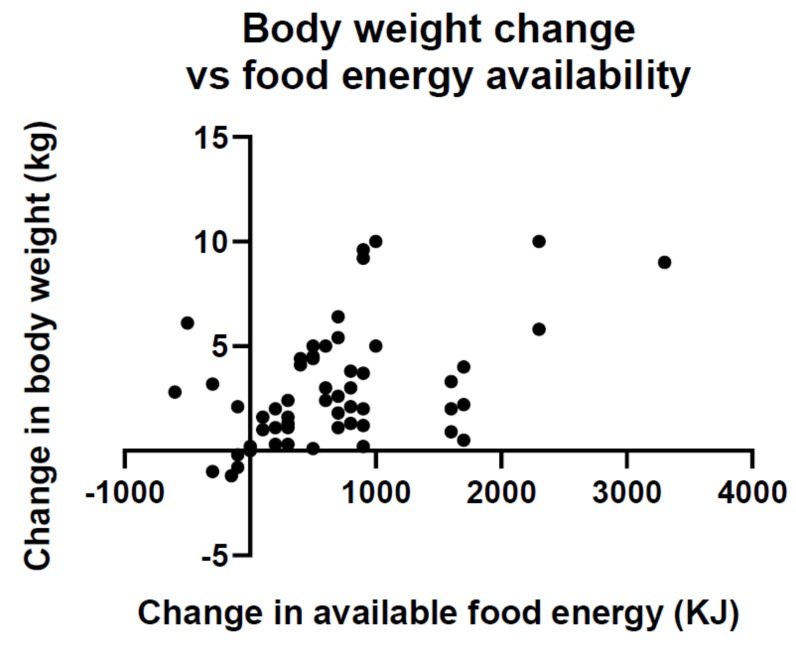
Change in average body weight for 69 countries as a function of changes in food energy supply. Adapted from Vandevijvere et al., 2015.

*Point 4. Economic growth and development brings about an overabundance of inexpensive foods that foster overconsumption and weight gain*. A byproduct of booming economic growth is an overabundance of moderately priced food. A global analysis has found that in 69 countries an increase in food energy supply over a 4 year interval was associated with a significant increase in average body weight (Figure 10) [105,106]. The magnitude of association was sufficient to explain population weight gain in the past 50 years.

*Point 5. The food industry is guided more by profit motive than by considerations of human health in aggressively promoting high-density palatable foods*. The food in US and developed countries was traditionally produced locally for neighborhood markets and with relatively little processing. Current system involves global suppliers to maximize efficiency, reduce costs, and increase production and profit before the food reaches the consumer. Supermarkets and the growing fast-food industry have introduced a variety of energy-dense processed foods, sometimes called ultra-processed foods because of their high content of palatable sugar and saturated fats. This is becoming a major source of energy in developed and developing countries and is seen as a driver of the obesity epidemic [107,108]. Spiking of processed food with additional sugar, fat, and salt increases its palatability, quantity eaten, and marketability, and is associated with development of psychological dependence or even addiction to such food. Eating such food is also associated with a number of health pathologies [107]. Ultra-processed food increases the risk of overeating not only because of its palatability, but also because of its high caloric density reflecting human dependence on ingested food volume for satiation, and not its caloric density. Of 22,659 adults whose caloric intakes of processed food and weight changes were monitored over a 5 year period by UK Biobank, 947 were obese and 1900 had abdominal obesity [108]. Participants in the highest quartile of ultra-processed food consumption had a 79% higher risk of developing overall obesity, 31% higher risk of experiencing a ≥5% increase in BMI, 35% increased risk of increased waist circumference, and 30% increased risk for abdominal obesity. They also had a 14% increased risk of greater percent body fat than individuals in the lowest quartile of consumption. It is estimated that factors leading to poor diet produce a bigger health burden than tobacco, alcohol, and inactivity put together [109]. Yet, influencing the food industry to change its profit-centered advertising and selling of ultra-processed food remains difficult [5]. The argument promoted by the food industry against regulation over its business strategies has focused on insisting that individuals are responsible for conscious control over their food choices [110,111].

### Suggestions for Counteracting Detrimental Aspects of Features of Developed Societies

**Figure 11:**
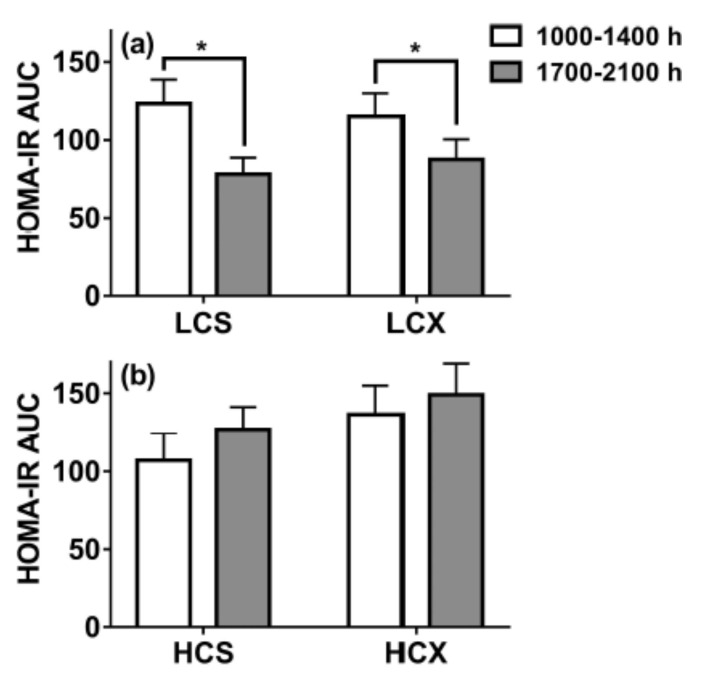
The effect of meals containing 30% carbohydrate (LC (**a**) panel) on the HOMA-IR measure of insulin resistance compared to 60% carbohydrate meals (HC (**b**) lower panel lower panel). The effect was evident in sedentary (S) as well and in exercise (X) trials before the meals. From Lin and Borer, 2016. * Indicates a significant difference.

Some societal obstacles to overeating such as characteristics of built environment we live in, global overabundance of relatively inexpensive food, misguided government dietary recommendations, and profit-driven strategies of the food industry to sell energy-dense unhealthy foods, cannot be easily changed but can be controlled if individuals are armed with helpful information. Two sets of research findings can help overcome or mitigate the damage done by misguided dietary recommendations that are likely to increase insulin resistance, the key consequence of overweight and obesity. Using behaviors that reduce insulin resistance is a powerful tool to counteract societal conditions that promote weight gain. The first such behavior entails a simple dietary manipulation [19]. By reducing the dietary content of carbohydrates from 60% to 30% of nutrient content leads to greater than 30% decline in the postprandial insulin responses and insulin resistance. The effect is achieved after the third daily exposure to reduced carbohydrate meals which in this study took place at 1700–2100 h (Figure 11). 

**Figure 12:**
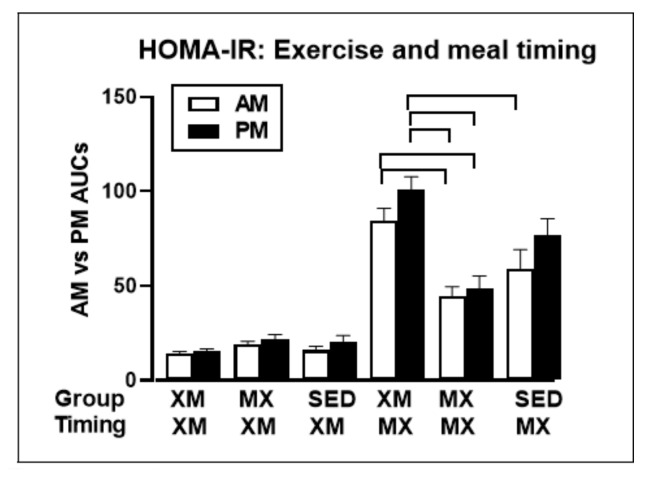
Exercise within an hour before eating (XM) increases HOMA-IR measure of insulin resistance, whether the exercise was done in the morning (open bar) or in the afternoon (solid bar). Exercise performed within an hour of eating a meal (MX) lowers insulin resistance by approximately 50% compared to exercise before eating. From Borer et al., 2021.

The second way to avoid weight gain or counteract development of insulin resistance as a consequence of being overweight or obese is by appropriate timing of exercise and meals [49]. While two hours of moderate-intensity exercise within an hour before eating a meal increases insulin resistance (Figure 12, XM, MX), the same exercise performed one hour after eating (MX MX), lowers insulin resistance by approximately 50% (Figure 12). This then provides a second deliberate behavior that can reduce insulin resistance and risk of pre-diabetes, and T2D in overweight and obese subjects in addition to a simple change in their macronutrient intake [19] (Figure 11). It simply involves appropriate timing of exercise with respect to meals [49] (Figure 12).

Beyond these two suggestions, some technological advances also can be utilized to prevent obesity. The single most significant and helpful technological advance for potential obesity management has been the access of the large proportion of American population to internet and smartphones. This has opened a new era in the use of the internet platform for both individual weight-control applications and for interventions aimed at weight loss [112]. Machine learning and speed of health-information delivery of relevant health variables by digital tracking devices can effectively motivate individuals to pursue healthy behaviors in contrast to ineffective delays and temporal discontinuities provided by traditional medical and instructional obesity interventions [113]. Some examples of successful suppression of overeating by electronic include presentation of ideal body images and relevant text [114]. Women who viewed slides depicting images of slender female models and exercise-related congruent text, and men who viewed slides depicting images of muscular male models, reduced the amount of food consumed. In a similar vein, men and women viewing a computer screensaver showing three of the famous skinny human-like sculptures by Alberto Giacometti, consumed less chocolate than when they were exposed to a more neutral work of art. Women, more than men, reduced their food intake when they were asked to indicate their body weight before chocolate tasting [115].

Relative to computer and smartphone-based approaches to weight control, a very simple device, a bathroom scale, has an outsize effect in curbing weight gain [116]. Its efficacy has been documented in late 1980s and subsequently in 1990s [117] when it was reported that a majority of people registered in National Weight Control Registry, who lost 30 lb and maintained this weight loss for an average of 5 years, weighed themselves several times a week. Combining daily weighing against a chart documenting weight-loss progress led not only to a weight loss, but also prevented weight regain [118].

## 6. Summary and Conclusions

The intent of this review is to survey four sets of obstacles to the control of eating and body weight maintenance and to provide information and insights on how to prevent or mitigate them. The first message attempted to fill the knowledge gap about how insulin resistance, the key defect resulting from overweight and obesity, develops and precipitates health pathologies. Second, six aspects of human physiology that support our tendency to overeat and gain weight easily, but have difficulty losing it, are described. Among those are an absence of feedback control against gaining weight, a non-homeostatic relationship between motivation to be physically active and weight gain, and dependence of hunger and satiation on the volume of food ingested by mouth and processed by the gastrointestinal tract and not on circulating metabolites or putative hunger and satiation hormones. Further, overeating and binging can increase stomach size and thus attenuate satiation. Almost any reduction in body weight is counteracted by reductions in resting metabolism, increased hunger, and enhanced efficiency of energy storage. The final evolutionary burden is the extraordinary human capacity to store body fat. As these genetically programmed obstacles cannot be directly overcome, our defenses include attentiveness to gastrointestinal signals of hunger and satiation and avoidance of overeating and excessive weight gain [13]. Third, four psychological tendencies that foster overeating are human craving for palatable and savory food, tendency to eat unnecessary meals over the extended wakeful period, social facilitation of food consumption in company of others, and human gullibility to overeat when offered more food with, or without, financial incentives. These psychological barriers can be overcome by conscious awareness of the risk of overeating palatable food, large portions, and overconsumption during celebrations [119]. A more effective control against unnecessary chaotic eating pattern is to time-restrict eating to a short period within 6 and 10 h. The fourth factor that abets weight gain includes five characteristics of developed societies. They include appliances and machines powered by fossil fuels as a substitute for human physical labor, design of built environments that necessitates using cars and public transportation instead of walking and bicycling, misleading government advice on the macronutrient intake that is associated with greater weight gain and insulin resistance, an overabundance of easy access to inexpensive food, and profit-driven efforts by the food industry to promote sales of energy-dense and nutritionally compromised foods. Solutions to some of these societal problems require some thoughtful individual choices, including reducing the carbohydrate content of meals to reduce insulin resistance, attempts to influence social policies, but also exploiting exercise not only for increases in energy expenditure but also for its effectiveness, when appropriately timed with respect to meals, to reduce insulin resistance [49,120]. Ultimately, success in preventing or mitigating overeating, fostering weight loss, and becoming more active requires a better understanding of the factors that stand in our way [13].

## Figures and Tables

**Table 1:** Differences in labor-saving aspects of Old Order Amish and contemporary lifestyles.

Tasks	Old Order Amish Way	Contemporary Way
Transportation	Horse and buggy, Walking	Car, public transit, some active transportation
Food provision	Farming, gardening, milking cattle by hand	Convenience and grocery stores, some gardening
Food preparation	Home cooking with wood-burning stoves	Restaurants, ready-to eat food, some home cooking
Food storage	Ice blocks cut from frozen lake ice	Refrigerators and freezers
House cleaning	Brooms and mops	Vacuum cleaners
Laundry	Hand washing, wringing, and air drying	Washing machines and dryers
Heating	Chopping firewood	HVAC
Mowing grass	Scythe, hand mowers	Motorized lawn mowers
Removing fallen leaves	Hand raking	Motorized air blowers
Spiritual life and Socializing	Church, mutual communal assistance	Television, internet, social media, some church

## Data Availability

As a review paper based on 122 sources, data cannot be easily retrieved.
